# Influence of Region on Sensory and Chemical Profiles of Pennsylvania Grüner Veltliner Wines

**DOI:** 10.3390/foods10040825

**Published:** 2021-04-10

**Authors:** Stephanie T. Keller, Andrew D. Harner, Michela Centinari, Ryan J. Elias, Helene Hopfer

**Affiliations:** 1 Department of Food Science, College of Agricultural Sciences, The Pennsylvania State University, University Park, PA 16802, USA; stk5117@psu.edu (S.T.K.); elias@psu.edu (R.J.E.); 2 Department of Plant Science, College of Agricultural Sciences, The Pennsylvania State University, University Park, PA 16802, USA; adh5238@psu.edu (A.D.H.); mzc22@psu.edu (M.C.); 3 Sensory Evaluation Center (SEC), The Pennsylvania State University, University Park, PA 16802, USA

**Keywords:** Grüner Veltliner wine, regionality, descriptive analysis, compositional analysis

## Abstract

The influence of cultural and environmental factors on the sensory and chemical profiles of wines has been the subject of research investigation for many years, and an examination of these relationships can help determine whether wine regional trends exist. The present study investigated the chemical and sensory factors that drive regional differences in Pennsylvania Grüner Veltliner wines through a controlled winemaking study across two vintages in 2018 and 2019. Descriptive analysis was used to identify key sensory attributes of Pennsylvania Grüner Veltliner. Intensities of these attributes were evaluated in wines vinified under identical conditions from grapes harvested across nine Pennsylvania vineyards. Chemical profiles of finished wines were examined through volatile, phenolic, and color analyses. Significant sensory differences were found between wine regions, with some trends consistent across both vintages; however, regionality based on compositional analyses was less clear. As the first study to examine Pennsylvania Grüner Veltliner wines sensorially, results revealed sensory characteristics that can be useful for wineries and their tasting room staff in marketing these lesser-known white wines to wine consumers as the variety grows in popularity in the state.

## 1. Introduction

Environmental conditions, in combination with many other factors, are known to affect final wine quality. Seasonal weather patterns, along with cultural factors such as soil type and grape variety, impart specific characteristics to wines [1]. This concept has been popularized in Old World wine regions as *terroir*, a term that refers to the geographical influence a place has on wine produced there [2]. While regional characteristics in wines are well recognized in Old World countries, New World wine regionality is less commonly recognized, but still an important concept, as wines produced in these regions become more renowned among the industry and consumers [3].

Regionality is a term used in the wine industry to describe the particular style of wine that a growing region produces [3]. Determining the characteristics of specific regions can help growers predict the quality of wine grapes before they are harvested and eventually vinified. Numerous studies have compared wine regions in a number of countries and found differences in aroma, flavor, and mouthfeel aspects in both commercial wines and wines that were made with a standard protocol to eliminate stylistic differences [4,5,6,7]. Some of these studies also found differences in the chemical profiles of investigated wines, or were able to relate aroma and flavor differences to environmental conditions [4,5,6,7].

While it may be expected that grapes grown thousands of kilometers from each other will yield wines with noticeably different characteristics, differences can also be found within one country of origin. In Australia, analysis of commercial Cabernet Sauvignon reported that the presence of specific volatile and non-volatile compounds was region-specific, including 2-isobutyl-3-methoxypyrazine, monoterpenes such as menthone and monomeric anthocyanins [8]. In Chile, regional proximity to the Andes Mountains negatively impacted ester (e.g., isoamyl octanoate, ethyl phenyl acetate, and ethyl dodecanoate), and acid concentration (e.g., hexanoic and octanoic acid) in commercial Carignan wines [9]. Studies in Canada have examined differences within a designated viticultural area, namely the Niagara Peninsula. Differences in aroma and flavor exist between sub-appellations of the region, both in commercial and research wines [10,11].

Examining regionality in wine-growing areas that are lesser known may help new or local regions gain recognition among wine consumers. In Pennsylvania, a mid-Atlantic state in the northeast United States and the location of the present study, wine regionality is only just beginning to be examined, and regional profiles for Pennsylvania wines have yet to be elucidated. Defining regional characteristics for the Pennsylvania wine industry can be useful in marketing specific characteristics of wines from a designated region and for building a reputation for high quality wine produced in that area. Researchers have examined two varieties of commercial Pennsylvania white wines for regional trends and found wines from the northwest region of the state to be sweeter; however, winemaker style likely influenced final wine composition and therefore masked potential regional characteristics [12].

Unlike other wine-growing areas, Pennsylvania does not have a “signature” variety; however, there are some grape varieties that are grown in many regions across the state, including Grüner Veltliner (Grüner V.), a *Vitis vinifera* white fleshed wine grape variety native to Austria. The variety was first brought to Pennsylvania in 2003 and has since become popular among growers in many of Pennsylvania’s wine-growing regions [13]. While Grüner V. has not been the subject of many studies in the United States, the variety has been studied more frequently in Austria, where it comprises 31% of the country’s total wine-growing area [14]. Because the variety is grown throughout the country’s wine regions, Grüner V. has been useful in examining regional differences of Austria’s wine-growing areas, specifically with the Weinviertel and Wagram regions [15,16].

Here, we characterize the chemical and sensory factors that drive regional differences in Pennsylvania Grüner V. wines through a controlled winemaking study over two consecutive growing seasons.

## 2. Materials and Methods

### 2.1. Site Selection

**Figure 1:**
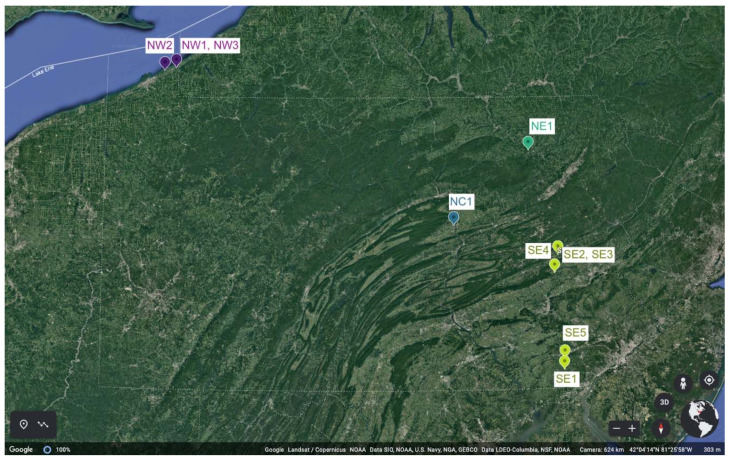
Location of vineyard sites within recognized regions of the Pennsylvania Winery Association (PWA; pennsylvaniawine.com, accessed on 10 April 2020). Vineyards with the same color belong to the same region.

Nine vineyards in four Pennsylvania wine regions defined by the Pennsylvania Winery Association (PWA) were chosen for the experiment in 2018 (Year 1) based on climate variability and representativeness of the number of wineries in the region that currently produce Grüner V.: two sites in the Northwest (NW), one in the North Central region (NC), one in the Northeast (NE), and five in the Southeast region (SE) (Figure 1 and Table S1). Canopy management practices were standardized to minimize confounding effects from vine management practices during the growing season. Specifically, shoot number was standardized to 10–14 shoots per linear m cordon, and fruit-zone leaf removal was implemented on the morning-sun side of the canopy following fruit-set both years. A HOBO^®^ weather station and datalogger (Onset Computer Corporation, Bourne, MA, USA) recorded vineyard air temperature and rainfall data in 15 min intervals during both growing seasons (1 May to 31 October 2018 and 2019) at all sites except NW2 and NC1; at these sites, weather data were collected from on-site stations associated with the Network for Environment and Weather Applications (NEWA; http://newa.cornell.edu, accessed on 10 April 2020). Vineyard air temperature was converted into growing degree days (GDD) using 10 °C as a baseline (GDD = ((maximum temperature + minimum temperature)/2)-10) (Table S1). Data were quantified in terms of véraison to harvest (_V) and total growing season (_T).

Significant bud damage occurred at multiple sites in 2019 (Year 2) due to below-average temperatures in the winter between the two growing seasons, resulting in the removal of NE1, SE4, and SE5. In Year 2, there were therefore two harvests at site NW1, with the second harvest completed on a later date and treated as a separate site (NW3). It should also be noted that sites SE2 and SE3 were located at the same commercial vineyard; however, grapes for these sites came from different vineyard blocks of the property.

### 2.2. Vinification

An average of 91.0 kg of fruit was hand harvested at each site and transported to the Department of Food Science at The Pennsylvania State University (University Park, PA, USA) where it was stored for less than 48 h at 4 °C before processing. After crushing and destemming, must was pressed to a yield of 50.08 L of juice per 100.0 kg of grapes using a bladder press. Juice was treated with Cinn Free (Scott Laboratories, Petaluma, CA, USA), a pectinolytic enzyme, at a rate of 15 mL/L. Juice was also treated with SO_2_ in the form of potassium metabisulfite (KMBS; Presque Isle Wine Cellars, North East, PA, USA) prior to cold settling; adjustment rates were dependent on cluster rot assessment and ranged from 30 to 50 ppm in Year 1 and 30 to 40 ppm in Year 2.

After cold settling overnight, juices were racked and chaptalized with sucrose to achieve a final total soluble solids (TSS) level of 22 °Brix across all treatments. If a given juice was above pH 3.4, its pH was adjusted to 3.4 using tartaric acid (Presque Isle Wine Cellars, North East, PA, USA). Similar adjustments have been made in prior wine regionality work [11,17,18]. In this study, we based the winemaking protocol on common PA winemaking practices for Grüner Veltliner, where chaptalization and pH adjustment are common. In addition, several studies on wine regionality also standardized final ethanol content through juice chaptalization (e.g., [11]), and some also acid adjusted (e.g., [17]). As demonstrated by Pineau et al. [18], juice chaptalization of Sauvignon blanc wines did not significantly affect wine aroma attributes across three harvest dates (except for stonefruit aroma in harvest 1, sweetness, acid balance, body, and length) compared to non-chaptalized wines. Furthermore, wine typicality ratings were mainly affected by grape ripeness rather than sugar adjustment in that study. We therefore decided to chaptalize juices as from experience we knew that the different sites differed in grape soluble sugar content. As ethanol is a significant factor in sensory perception of wines (e.g., [19]), we aimed to target equivalent final ethanol contents in all wines across both years.

The adjusted juices were racked into two 5 gallon (18.93 L) glass carboys prior to inoculation with yeast. Due to low juice yields for some sites in Year 1, two experimental sites were fermented in 1 gallon (3.79 L) glass jars and one site was fermented in 3 gallon (11.36 L) carboys. In Year 2, one of the 7 sites was fermented in 3 gallon carboys. Each fermentation replicate was inoculated with *Saccharomyces cerevisiae* EC-1118 yeast (Lallemand, Petaluma, CA, USA) at a rate of 0.25 g/L with an addition of 0.30 g/L Go-Ferm nutrient (Lallemand). Fermentations were temperature controlled in Year 1 using glycol jackets (Gotta Brew, Sacramento, CA, USA) maintained at 15 °C and in Year 2 using a chilled water bath system with a glycol chiller set to 12.8 °C. In both years, fermentation temperatures were both typical of white wine fermentation [20,21] and in alignment with Grüner Veltliner fermentation practices in PA [22].

Yeast assimilable nitrogen (YAN) adjustments were performed 24 h post-inoculation and at one-third sugar depletion using Fermaid K nutrient (Lallemand) to achieve a final concentration of 0.25 g/L. Alcoholic fermentation was monitored daily by TSS readings via hydrometry. When TSS values measured below 0 °Brix, AimTab Reducing Substance tablets (Germaine Laboratories, San Antonio, TX, USA) were used to confirm dryness of wines, defined as ≤1% residual sugar. Fermentations were held at 4 °C for cold settling, subsequently racked off lees, and treated with the enzyme blend Scottzyme KS (Scott Laboratories) at a rate of 79.25 µL/L. SO_2_ was adjusted to achieve 0.85 mg/L molecular SO_2_. Replicates were manually bottled in 375 mL green glass bottles and sealed with Saranex-lined Stelvin screw cap closures. Bottled wines were stored at 3 °C until sensory evaluation and chemical analysis (within three months in each year).

### 2.3. Descriptive Analysis

Descriptive analysis protocol was evaluated by The Pennsylvania State University Institutional Review Board (protocol #STUDY00008551) and found to be exempt under category 6 (Taste and Food Quality Evaluation). A panel of 8 participants (4 females; ages 24–60) was trained to evaluate Year 1 wines three months after bottling. In the second year, the panel consisted of 9 individuals (6 females; ages 32–60), 6 of whom were on the previous year’s panel. Participants were selected based on previous alcoholic beverage panel experience conducted at the Sensory Evaluation Center (SEC) at The Pennsylvania State University. In Year 1, panelists were trained for 14 h over a 4-week period on orthonasal aroma, flavor (in-mouth aroma), taste, and mouthfeel attributes found in the wines using generic descriptive analysis (DA) [23]. Panelists generated terms and agreed upon corresponding physical and verbal references (Table 1) for each attribute in the training sessions. Attribute terms were generated by blindly exposing the panelists to the different wines, with each wine replicate presented at least once during training. The panel developed a list of two appearance, 15 orthonasal aroma, 5 taste, three mouthfeel, and 15 in-mouth flavor attributes (Table 1). Three of the attributes (citrus aroma/flavor, other fruit aroma/flavor, and sour taste) used two reference standards to describe the term. After the attribute list was generated, panelists were trained on attribute rating and scaling. Intensities of each attribute were rated on line scales that had anchor terms on both ends. While wine and other beverage samples can be evaluated in black glasses to eliminate physical evaluation, visual differences among samples were frequently discussed in panel training, and so two appearance attributes were included in evaluation. Appearance attributes (yellow color and haziness) were anchored with the terms “No (yellow/haze)” at the left end of the scale to “Very (yellow/hazy)” at the right end. Aroma, taste, mouthfeel, and flavor attributes were anchored with “None” at the left end of the scale to “A lot” at the right end.

Blind duplicate samples were used to evaluate panel performance and rating agreement. Panelists were considered trained when they could rate blind duplicate samples and find no significant differences (*p* < 0.05) between them using analysis of variance (ANOVA). Fourteen of the 18 wine samples (=9 sites × 2 fermentation replicates) were evaluated in sensory triplicate, while four samples were evaluated in sensory duplicate due to low sample volume. Panelists evaluated samples in 7 sessions over a period of three weeks. Evaluation was conducted in individual booths in the SEC under white light and positive pressure and results were collected using Compusense Cloud software (Academic Consortium, Guelph, ON, Canada). Panelists were presented with 30 mL of each sample at room temperature in clear ISO certified tasting glasses covered with clear plastic Petri dishes. Each panelist evaluated 6 samples per session that were labeled with randomized three-digit blinding codes and presented in a modified Williams Latin square block design. Panelists were prompted to cleanse their palate with water and unsalted crackers (Mondelēz Global LLC, East Hanover, NJ, USA) during a forced 60 s break between samples. One panelist missed two evaluation sessions, and the missing values were imputed using the panelist’s mean replicate values.

In the second year, the panelists were trained for 19 h on the same reference standards used in the Year 1 DA. The panel was given the option to add references to this list; after being presented with all replicates of Year 2 wines, the panel determined additional references were not needed. Panelists were deemed trained when they evaluated blind duplicate samples and found no significant differences between them. Sample evaluation took place over 5 sessions in which panelists evaluated samples in sensory triplicate using Compusense Cloud software, with fermentation replicates treated as separate samples. Evaluation conditions were identical to Year 1, with the following exceptions: panelists were presented with 10 samples per session for the first four sessions and evaluated 5 samples during the last session. Panelists received 5 samples at a time with a forced 60 s break between samples, and a forced break of 10 min between two sets of 5 samples.

### 2.4. Chemical Analysis

#### 2.4.1. Wine Chemical Analysis

Wines were sampled prior to bottling for basic wine chemical analysis via Fourier-transform infrared spectroscopy (FTIR), including residual sugar (RS), ethanol content, volatile acidity (VA), free and total sulfur content, titratable acidity (TA), pH, lactic acid, and malate content, by the Cornell Craft Beverage Analytical Laboratory (Geneva, NY, USA).

#### 2.4.2. Volatile Wine Analysis

Headspace–solid-phase microextraction–gas chromatography–mass spectrometry (HS–SPME–GC–MS) was used to determine aroma composition of wines using a method from previous work, with some modifications [24,25]. An Agilent Technologies 7890B GC 5977B MS (Santa Clara, CA) with an MPS autosampler (Gerstel, Inc., Linthicum, MD, USA) and Rtx-Wax 30 m × 0.25 mm × 0.25 µm GC column (Restek, Bellefonte, PA, USA) was used for sample analysis. Agilent MassHunter GC/MS software and Gerstel MAESTRO software was used to control the GC and autosampler, respectively. For sample preparation, 2 mL of wine and 50 µL of internal standard mix (9.9 mg/L d8-napthalene and 13.7 mg/L 2-octanol in 100% HPLC grade methanol) were added to 20 mL crimp cap headspace vials (Restek) containing 2 g of sodium chloride (Dot Scientific, Burton, MI, USA). Samples were incubated at 30 °C for 5 min and then extracted for 30 min at the same temperature using a 2 cm DVB/CAR/PDMS SPME fiber (Supelco, Bellefonte, PA, USA). The fiber was desorbed at 250 °C for 10 min in the splitless mode, using a constant helium flow of 1 mL/min for analyte separation throughout the run and an oven program starting at 30 °C for 1 min, followed by a 10 °C/min ramp up to 250 °C and a hold for 5 min at this temperature. The analytes were detected in the scan mode from 33 to 350 amu with 8.1 spectra collected per second.

Sampling occurred during the DA evaluation sessions so analysis could be completed on sensory samples and multiple bottles. For wines made in 2018 (Year 1), samples for aroma analysis were prepared in 6 replicates (2 replicates from each sample evaluation day), and for wines made in 2019 (Year 2) samples were prepared in 5 replicates (1 replicate from each evaluation day). Compounds were identified via the National Institute of Standards and Technology (NIST) Mass Spectral Library (version 17). Compounds were reported in internal standard (IS) equivalents (µg/L).

#### 2.4.3. Phenolic Analysis of Wine

Individual phenolics were quantified using high performance liquid chromatography (HPLC) with a method adapted from Sheridan and Elias [26]. A Shimadzu HPLC (Shimadzu, Torrance, CA, USA) equipped with LC-10ADvp pumps, a reverse phase Eclipse Plus C18 column (4.6 × 250 mm, 5 mm; Agilent Technologies), SPD-M10Avp controller, DGU-14A degasser, SCL-10Avp UV-DAD detector, SIL-20AC HT autosampler, and Brinkman CH-30 column heater with an Eppendorf TC-45 temperature controller was used for analysis. The binary mobile phase system consisted of 1% phosphoric acid in water (A) and methanol (B). Samples were centrifuged at 13,500 × *g* for 5 min (Microfuge 16, Beckman Coulter, Indianapolis, IN, USA) and 200 µL of sample was transferred into 11 mm glass HPLC vials (VWR, Radnor, PA, USA) with 250 µL reduced volume vial inserts (6 × 31 mm; MicroSolv Technology Corporation, Leland, NC, USA). Injection volume was 20 µL, flow rate was 0.7 mL per minute, and column temperature was 30 °C. Phenolics were eluted using the following sequence: 0–5 min, 5% B; 7 min, 15% B; 10 min, 17.5% B; 22 min, 19.75% B; 26–33 min, 50% B; 34–35 min, 85% B, and re-equilibration at 5% B for 7 min before the next injection. For phenolic analysis, gallic acid, (+)-catechin, and (−)-epicatechin were quantified in wine samples at 210 nm (280 nm served as the qualifier wavelength), based on previous work in identifying phenolic compounds in white wine and juice [27]. External 8-point calibration curves were established between 0 and 10 mg/L.

#### 2.4.4. Color Analysis of Wines via CIE-Lab Color Measurements

Chromatic characteristics of wine samples were obtained following the protocol of the Compendium of International Analysis of Methods of Wine and Must Analysis [28]. Samples were centrifuged at 2500× *g* for 5 min and transferred into 10 mm glass cuvettes. Transmittance of the sample was measured every 5 nm from 380 to 780 nm, with Illuminant D_65_ and the 10° Observer used as standard conditions. Ultrapure water was used as a blank and samples were measured in triplicate. Transmittance values were converted to the colorimetric coordinates L*, a*, and b*, where the L* parameter represents lightness (from 0 = black to 100 = white), a* represents the green (−a*)—red (+a*) axis, and b* the blue (−b*) to yellow (+b*) dimension [29].

### 2.5. Statistical Analysis

Statistical analyses were performed using RStudio software (version 1.1.463, Boston, MA, USA). For analysis of sensory data, ANOVA was completed with wine, judge, fermentation replicate and all two-way interactions to determine significant attribute differences among the samples with wine, judge, and fermentation replicate treated as main, fixed effects. A pseudo-mixed model was used to calculate F-ratio for samples that had significant wine-by-judge interaction in addition to a significant wine effect [30]. ANOVA with region as the main effect was then used in combination with Least Square Means (LSMeans) post hoc comparison to examine potential regional differences in the attributes, using the emmeans package (version 1.4.6) [31]. The SensoMineR package (version 1.23) [32] was used for principal component analysis (PCA). Ninety-five percent confidence ellipses (CE) were calculated by the panellipse.session function in the SensoMineR package, with fermentation replicate being used as the session factor.

Statistical analysis for basic wine chemical analysis was completed using one-way ANOVA with wine as a main factor. To examine potential regional differences, ANOVA was conducted with region as the main factor, with the agricolae package (version 1.2-8) being used for Tukey’s post hoc comparison [33]. PARADISe software (version 3.1) [34] was used for analysis of the volatile compounds. One-way ANOVA was used to determine whether significant differences in concentration for the compounds existed between wine samples and between regions.

Weather parameters including rainfall and GDD were included as supplementary variables in a PCA of the chemical and sensory data using the SensoMineR package. Significance for all analyses was defined as *p* < 0.05.

## 3. Results and Discussion

### 3.1. Descriptive Analysis

In the first year (wines made in 2018), the trained panel found the wines to differ significantly in both appearance attributes, three aroma attributes (earthy, sulfur, and thiol), two taste and mouthfeel attributes (sour taste, warm/hot mouthfeel), and two flavor attributes (thiol and ethanol). There was a significant fermentation replicate effect for haziness and sulfur aroma. The significant haziness effect was attributed to excess sulfur additions to SE1 wine replicates, based on false low readings from aeration oxidation, and so this attribute was not included in the PCA.

Among the significantly different attributes, only yellow color and haziness were different by region in Year 1 (Table S2). Wines from the NW region were rated significantly higher in yellow color than the other three regions (5.52 vs. 2.51 (SE), 2.87 (NC), and 4.20 (NE) on a 10-point scale; Table S2). Additionally, wines from the NE region were significantly higher in yellow color than those from the SE and NC regions (4.20 vs. 2.51 (SE) and 2.87 (NC)). Grape maturity at harvest may have affected the resulting wine color, with more mature grapes producing wines that were more yellow in color. Previous work on white wines found grapes harvested at an earlier date to be lighter in color and less intense, as indicated by higher L* and lower C* and b* values [35]. NW2 and NE1 were the last sites to be harvested and had two of the highest TSS levels (19.6 and 16.4 °Brix, respectively) (Table S3), indicating these sites provided more mature grapes for vinification compared to sites harvested earlier at lower TSS levels. Weather effects may have also contributed to the regional color differences. Elevated temperatures could contribute to greater accumulation of phenolic compounds that influence wine color. Increased daytime temperatures in the vineyard can increase berry color ratings in white *vinifera* varieties, which could affect final wine color [36]. However, we tend to exclude that differences in GDD across sites explained regional trends in wine color alone in this study because cumulative GDD (GDD_T) was lowest for the NE and NW regions compared to the other wine regions (Table S1). Furthermore, GDD during fruit ripening (GDD_V), which tend to be more important for phenolic compounds accumulation, did not differ markedly across sites, except for NC1. It is possible that differences in temperature may have not been large enough to result in significant color differences in finished wines. In addition, fruit sunlight exposure may have also played a role in terms of final wine color.

**Figure 2:**
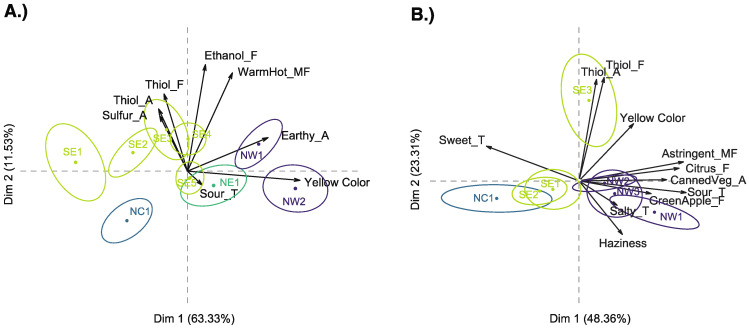
Principal component analysis (PCA) biplot for the significant wine sensory attributes of Year 1 (made in 2018) (**A**) and Year 2 (made in 2019) (**B**) Grüner V. wines. Wines are shown with 95% confidence ellipses and colored by region. Attributes ending in A indicate orthonasal aroma, F indicate in-mouth flavor, MF indicate mouthfeel, and T indicate taste attributes.

All 7 significant wine sensory attributes were used in the PCA, with the exception of haziness which was a result from excess SO_2_ addition to SE1 wines. The PCA (Figure 2A) captured 74.86% of the total variance in the first two dimensions, and consistent regional trends among the wines were observed. The NC sample, located in the bottom left quadrant of the biplot, separated from all other wine samples. While the two NW wines are significantly different from each other, as indicated by the 95% CE not overlapping, the wines were both positively loaded on PC 1. All 5 SE wines, except for SE5, were positively loaded on PC 2, and three of the 5 samples (SE1, 2, and 3) were located on the negative PC 1 axis. SE5 and NE1 showed sensory profiles that were not significantly different as indicated by their overlapping confidence ellipses, while wines NE1 and NW2 were also not significantly different.

Yellow color was a clear driver of separation between wines from the different regions along the first dimension, PC 1 (Figure 2A). Wines that were rated highest in yellow color (NW1 and NW2) were positively loaded along the first dimension, while wines from the SE region, located along the negative PC 1 axis, were lowest in yellow color. Conversely, thiol aroma and flavor, ethanol flavor, and warm/hot mouthfeel as opposed to sour taste drove separation along the second dimension; the three samples which were rated highest in these attributes (SE1, 3, and 4), were positively loaded on PC 2, while the NC wine was rated highest in sour taste, and thus, located on the negative PC 2 axis. Sour taste showed a negative correlation to warm/hot mouthfeel, which was significantly loaded onto PC2, and ethanol flavor.

In Year 2 (wines made in 2019), the trained panel found the wines to differ significantly in both appearance attributes, three aroma attributes (canned vegetable, thiol, floral), four taste and mouthfeel attributes (sour, sweet, and salty taste, astringent mouthfeel) and three flavor attributes (thiol, citrus, and green apple; data not shown). There was a significant fermentation replicate effect for green apple flavor. Multiple attributes were significantly different in both years, namely yellow color, haziness, thiol aroma and flavor, and sour taste (data not shown). All of the significantly different wine attributes, with the exception of floral and canned vegetable aromas, also showed significant differences by region for Year 2 (Table S4). Wines from the NW region (n = 6) were again the highest in yellow color ratings and were rated significantly higher than wines from the NC and SE regions. NW wines were also rated as significantly less hazy than SE wines, which was consistent with Year 1 results. In Year 1, haziness and yellow color were negatively correlated to each other; however, these attributes were independent of each other in Year 2. NW wines were also rated highest in sour taste, astringent mouthfeel, citrus flavor, and green apple flavor, and differed significantly in sour taste, sweet taste, and citrus flavor attributes from the NC and SE regions.

While grape maturity likely contributed to higher yellow color ratings for NW wines in Year 1, fruit harvested from two of the three NW sites were least mature in Year 2 (see Table S3). However, since these wines had higher acidity, less SO_2_ was added to these wines after fermentation, as less sulfur dioxide was needed to achieve 0.8 g/L molecular SO_2_ at lower pH. Since there was less sulfur in these wines to act as an antioxidant, it is possible oxidation reactions caused a darker, more yellow color to form in these wines, resulting in panelists rating NW wines highest in yellow color for Year 2 despite the fruit being less mature.

The NC sample was highest in sweet taste and differed significantly from wines from the NW and SE regions. These ratings were expected, as the NW1 wine, followed by NW2 and NW3, had the highest TA values among the samples, while both fermentation replicates of the NC region wine had the highest RS values (Table S5). It is possible that panelsts rated NW samples highest in citrus and green apple flavors due to the association of sourness with these attributes, which is supported by a significant correlation (*r*(13) = 0.59, *p* = 2.79 × 10^−2^ for green apple flavor and sour taste; *r*(13) = 0.87, *p* = 5.82 × 10^−5^ for citrus flavor and sour taste). The three SE wines were rated significantly higher in thiol aroma and flavor than wines from other regions. This was again similar to the results from Year 1.

When examining the PCA biplot for Year 2 results (Figure 2B), similar sample separation trends were found compared to Year 1. All significant attributes were used in the PCA, and 71.67% of variation was captured within the first two dimensions. The three NW wines again grouped together in the bottom right quadrant. The NC sample was again located in the bottom left quadrant; however, this sample was not significantly different from the SE1 and SE2 wines as it was in Year 1. The last wine from the SE region, SE3, differed significantly from the other SE wines and was the only wine to be positively correlated to PC 2.

PC 1 captured 48.36% of the total sample variation. Both appearance attributes (yellow color, haziness), sour and salty taste, astringent mouthfeel, canned vegetable aroma, and citrus and green apple flavors were all positively loaded along PC 1. Sour and sweet tastes were negatively correlated with each other along PC 1, which is expected, as mixture suppression can cause sweet taste to be reduced in the presence of acids in food or beverages [37]. Another explanation for this could be the ripeness levels of the grapes used for making these wines—grapes used in vinifying NW1 and NW3 wines were the least ripe of all sites (harvested at 14.0 and 16.6 °Brix, respectively), which could explain some of the higher ratings for attributes associated with unripeness, such as canned vegetable aroma or sour taste. Research examining influence of fruit maturity in red wine varieties found that fruit harvested at lower TSS results in wines higher in vegetal aroma and sourness ratings [38,39]. Similar vegetal wine aroma differences were found in a white wine variety, Fiano, harvested at different maturity levels; however, this study compared normal harvest and late harvest maturity [40].

Separation along PC 2, which captured 23.31% of sample variation, was driven by thiol aroma and flavor ratings, which showed a positive correlation to PC 2. When examining the significant attributes along with the samples on the biplot, SE3 wine separated from the other wines, including those from the SE region, due to significantly higher ratings for thiol aroma. It should be noted that SE3 separated from SE2 in the PCA. Grapes for SE2 and SE3 wines originate from different vineyards at the same geographic site. This suggests that mesoclimatic differences can lead to aroma and flavor differences in Grüner V. wines. The two vineyards experience the same weather patterns, however, are located at different elevations (187 vs. 219 m above sea level). While this is a relatively small difference in elevation, air displacement can still vary among sites with relative elevation differences [41]. The vineyards also differ in row orientation (north–south for SE2 vs. east–west for SE3), which could impact fruit sun exposure and solar radiation intercepted by the canopy [42]. These factors could be the reason for the observed sensory differences in the wines, as winemaking was controlled in this experiment.

### 3.2. Chemical Analysis

#### 3.2.1. Wine Chemical Analysis

In Year 1 (wines made in 2018), regional differences were found with respect to wine pH, malate, lactic, and total SO_2_ content (see Table S5). Sulfur dioxide was added to wines based on pH to obtain 0.8 mg/L molecular SO_2_, and compositional differences in acidity likely caused this difference. In Year 2 (wines made in 2019), ethanol content, RS, pH, TA, malate, and free and total SO_2_ content significantly differed by region (see Table S5). NC wines were significantly higher in TA and lower in pH. This is likely due to gapes from the NW1 and NW3 site being less ripe at harvest than those from other sites. NC wines were higher in RS than those from other regions; however, since juices were chaptalized this difference is likely due to an incomplete fermentation rather than a regional difference. Since juices were chaptalized, the significant difference in alcohol content is also likely due to slight variation in fermentation dynamics. NW wines had lower free and total SO_2_ concentrations due to achieving 0.8 mg/L molecular SO_2_ at a low pH.

#### 3.2.2. Volatile Aroma Analysis

In Year 1 (wines made in 2018), a total of 33 compounds were detected, and 12 were found to differ significantly among all wine samples. A number of these compounds were fermentation-derived esters including ethyl decanoate, ethyl hexanoate, ethyl octanoate, as well as hexyl acetate and 2-hexen-1-ol acetate (Table S6). These compounds contribute to a general fruity aroma in wine and include odors of apple and peach [43]. Significant regional effects were found in six of these volatile compounds (Table S7). The NC region was highest in hexyl acetate, while ethyl hexanoate was higher in SE wines than in NC and NW wines, and 1-hexanol was found in higher concentrations in the NE region compared to the NC region.

In Year 2 wines, made in 2019, 51 compounds were detected and 20 volatile compounds differed significantly in concentration (Table S8). Fifteen of these 20 compounds also differed significantly by region in the second year (Table S9). SE wines had significantly higher concentration of the following compounds compared to the NW and NC regions: ethyl acetate, 1-propanol, butyl acetate, isobutanol, amyl acetate, 1-hexanol, acetic acid, and 2-hexen-1-ol. While some of these compounds are associated with apple aroma, namely butyl acetate and amyl acetate, SE1 and SE3 were the two wines with the lowest green apple aroma ratings, suggesting these compounds are not contributing to perceived apple aroma. Although chemical/solvent aroma was not significantly different between the wines, the three SE samples had the highest chemical/solvent aroma ratings among the samples, which could be caused by their higher concentrations of ethyl acetate. In contrast, SE wines were significantly lower in ethyl butyrate than NW wines. Wines from the NW region were lowest in 3-hexen-1-ol, which gives a grassy aroma in wines. NC wines were highest in methyl hexanoate and methyl octanoate, while NC wines were lowest in these compounds. Methyl octanoate can provide a range of odors including orange, herbal, and vegetable. Wines from the NW region were higher in canned vegetable aroma and citrus flavor, and it is possible methyl octanoate could be contributing to panelists rating these attributes higher in NW wines.

#### 3.2.3. Phenolic and Color Analysis

In Year 1, relative abundance significantly differed by wine for the three phenolic compounds examined. Relative abundances of (−)-epicatechin and (+)-catechin also varied by region, however, gallic acid abundance did not differ between regions (Table S10). Regional differences were likely found due to (−)-epicatechin being undetectable in NC wines, while (+)-catechin was not detected in NC1 and SE5 samples.

In Year 2 samples, relative abundance significantly differed for all compounds by wine; however, no compounds were significantly different by region (Table S10). NW2 was significantly higher in both gallic acid and (−)-epicatechin than all other wines. NW1 and NW3 wines were lowest in all compounds ((−)-epicatechin and (+)-catechin were not detected in these samples), which may be due to limited cluster thinning at these sites. Clusters per vine counts at these sites were high (54 clusters per vine in Year 2). Cluster thinning is used to improve grape quality and control crop load in over cropping vines, and phenolic levels can increase in grapes when cluster thinning is employed [44,45,46]. It is likely that lack of adequate crop thinning at the NW1 and NW3 sites altered phenolic composition and resulted in lower phenolic concentrations in these wines.

Although not significantly different by wine, the NW1 and NW3 samples still had the highest ratings of astringent mouthfeel in Year 2. Astringency can be caused by a number of compounds present in wines, including phenolics. However, high acidity can also lead to high astringency ratings in white wines regardless of total phenolic concentration [47]. NW1 and NW3 wines had the lowest pH and highest TA values of the samples, suggesting that astringency ratings were at least partially due to higher acidity levels and not concentration of phenolics.

Colorimetric coordinates L*, a*, and b* were used to examine color variation among wine samples. In Year 1 wines, there was variation in L* for samples regardless of region. All but four replicates of SE wines and all but one replicate of NW wines had L* values higher than 99.00. NE wines and one replicate of NC wines had L* values less than 99.00. a* values also varied within region for SE wines, ranging from −0.37 to −0.81. NW wines generally had lower a* values than wines from other regions, ranging from −0.78 to −0.88. A clearer regional trend was seen in b* values, with SE and NC wines having b* values less than 3.00 and NW and NE wines having b* values greater than 3.00. These results are consistent with visual sensory evaluation, in which the trained panel found NW and NE wines to be significantly higher in yellow color than wines from the SE and NC regions.

L* values generally similar across regions for Year 2, consistent with Year 1 results. All but 3 of 15 fermentation replicates had L* values measuring between 98.00 and 99.00. There was variation in a* measurements across wines, ranging from −0.40 to 0.03 for SE wines and −0.36 to −0.16 for NW wines. NW wines had the highest b* values ranging from 4.36 to 5.07. These results are consistent with visual sensory evaluation, in which the trained panel rated NW wines significantly higher in yellow color than other regions. The trained panel rated NC wines higher in yellow color than SE wines. However, b* values for NC wines were lower than those of SE wines.

### 3.3. Relating Sensory and Instrumental Data

**Figure 3:**
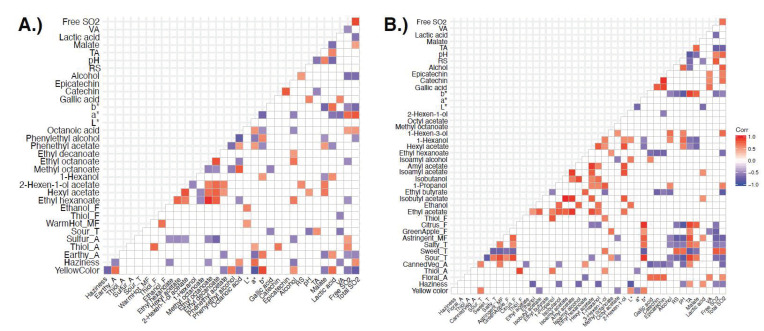
Significant correlation (*p* < 0.05) of sensory and chemistry parameters for Year 1 (**A**) and Year 2 (**B**) Grüner V. wines. Positive correlations are shown in red shades while negative correlations are shown in blue.

Significant sensory and wine instrumental variables were correlated using Pearson’s correlation coefficient. Statistical correlations may indicate potential relationships between instrumental and sensory data. Examining these correlations does not prove causality, however, it can give insight into which relationships should be further studied and which ones may be causal. Significant linear correlations between variables for both years are shown in Figure 3. Year 1 (Figure 3A) resulted in fewer significant linear correlations between sensory attributes and instrumental variables than Year 2 (*n* = 122 vs. *n* = 244), possibly because there were fewer significant differences between wines in sensory attributes and aroma compounds in Year 1.

For both years, yellow color was positively correlated with b* values (Year 1: *r*(16) = 0.88, *p* = 1.26 × 10^−6^; Year 2: *r*(13) = 0.64, *p* = 1.378 × 10^−2^) and negatively with a* values (Year 1: *r*(16) = −0.89, *p* = 7.23 × 10^−7^; Year 2: *r*(13) = −0.56, *p* = 0.04). Higher b* values indicate a sample is more yellow in color, so b* and yellow color correlation was expected. a* values were negative for the samples, which indicate a sample is greener and less red, and for white wines a negative a* value is not unexpected since there is limited skin contact to give a more orange or red color to wines. The strong positive thiol aroma and flavor correlation was consistent in both years as well (Year 1: *r*(16) = 0.75, *p* = 3.1 × 10^−4^; Year 2: *r*(13) = 0.89, *p* = 1.364 × 10^−5^). Sour taste was positively correlated with TA values and negatively with pH values in both years. This was also expected as lower pH values indicate higher acidity, while higher TA values also indicate higher acidity.

There were a number of significant correlations that were only found in the Year 1 data (Figure 3A). In Year 1, thiol aroma, i.e., the aroma associated with 4MMP (Table 1), was positively correlated to (+)-catechin (*r*(16) = 0.68, *p* = 0.002) and free SO_2_ (*r*(16) = 0.59, *p* = 0.01). It is possible that sulfur acted as an antioxidant and prevented oxidation of thiols and subsequent loss of thiol aroma. Research has shown that additions of SO_2_ can prevent loss of thiols in the presence of (+)-catechin in model wine [43]. Work by Saucier and Waterhouse has also found (+)-catechin to have a synergistic effect in combination with SO_2_ in preventing oxidation in model wine [48]. Sulfur aroma, i.e., the aroma associated with the smell of potassium metabisulfite (Table 1), was also positively correlated with free SO_2_ (*r*(16) = 0.49, *p* = 0.037). This was expected, as aroma of SO_2_ is detected when a high proportion of SO_2_ is in the free form.

There were several significant negative correlations between sensory and instrumental variables in Year 1 (Figure 3A). Sulfur aroma was negatively correlated with multiple esters, including ethyl hexanoate, hexyl acetate, 2-hexen-1-ol acetate, ethyl octanoate, and ethyl decanoate. Sulfur dioxide can bind with carbonyl compounds and reduce concentrations of aroma compounds such as β-damascenone or acetaldehyde [49]; however, in the case of volatile esters, additions of sulfur dioxide can retain ester concentrations after three months of aging [50], so it is likely this negative correlation is not causal. Gallic acid was negatively correlated with sour taste (*r*(16) = −0.54, *p* = 0.021) and positively correlated with pH (*r*(16) = 0.62, *p* = 0.006). Gallic acid can provide both bitter taste and astringent mouthfeel in wines [51], and it is possible higher concentrations of gallic acid in samples reduced perception of sour taste due to mixture suppression [37].

Correlations for Year 2 sensory and instrumental data are shown in Figure 3B. Thiol aroma was positively correlated with 1-propanol and 3-hexen-1-ol, which provide an alcohol and grassy aroma, respectively. It is unlikely that these compounds are contributing to the thiol aroma perceived in wines, as panelists were trained using a 4-MMP solution to define thiol aroma. Gallic acid and isoamyl alcohol, which provides a fruity aroma, were also positively correlated. Floral aroma was positively correlated with all three phenolic compounds, VA, and total SO_2_. Gallic acid, (−)-epicatechin, and (+)-catechin are not believed to impart aroma or flavor characteristics to wines, so the correlation with floral aroma is likely coincidental. As previously mentioned, additions of sulfur dioxide can decrease floral aromas by binding carbonyl compounds [49], so it is unlikely that SO_2_ bound to floral compounds in the wine samples since these two variables were positively correlated. Sweet taste was positively correlated with RS (*r*(13) = 0.76, *p* = 1.72 × 10^−3^), indicating panelist perceived wines with higher sugar content as sweeter than other samples. Green apple flavor was positively correlated with TA (*r*(13) = 0.59, *p* = 2.72 × 10^−2^). Higher TA values indicate a sample is more acidic, and it is possible panelists associated more sour samples with green apple flavor. This flavor attribute was also correlated with methyl octanoate, which can give a waxy and green aroma.

Haziness was negatively correlated with a number of volatile compounds in Year 2, including ethanol, isobutanol, isoamyl alcohol, and 1-hexanol. Haziness was also negatively correlated with L* (*r*(13) = −0.86, *p* = 8.89 × 10^−5^), indicating that the trained panel rated darker samples as hazier. Floral aroma was negatively correlated with ethyl hexanoate (*r*(13) = −0.62, *p* = 1.82 × 10^−2^), which can give a green apple aroma. Ethyl hexanoate was also negatively correlated with the three phenolic compounds quantified.

The most prominent difference in correlation results across years is that sulfur aroma was negatively correlated with many ethyl and acetate esters in Year 1 while esters were negatively correlated with haziness in Year 2. It is possible that higher SO_2_ levels in Year 1 wines provided a sulfur aroma that overpowered fruity notes provided by esters.

### 3.4. Relating Wine Data with Weather Conditions

**Figure 4:**
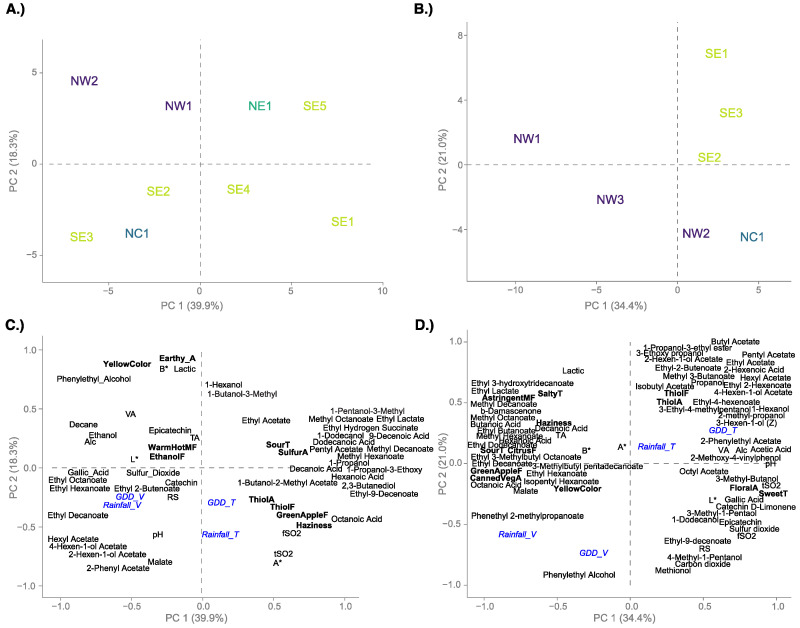
Principal component analysis (PCA) results for wine samples (**A**,**B**) and sensory and chemical variables (**C**,**D**) for Year 1 (**A**,**C**) and Year 2 (**B**,**D**) results. Rainfall and GDD data shown in blue were superimposed as supplementary variables onto the PCA and quantified in two ways: véraison to harvest (_V) and total (_T) values. See Tables S6 and S8 for variable loadings.

To gain insight on what may be driving regional differences in Grüner V. wines, weather data were added to the PCA as supplementary variables (Figure 4). In Year 1 (Figure 4A,C), 58.2% of variance was explained in the first two dimensions. In the samples plot, NW wines grouped in the second quadrant of the plot. SE2 and SE3, the samples from different vineyard plots of the same site, grouped in quadrant 3, as did the wine from the NC region. The remaining four samples were positively loaded on PC1, with NE1 and SE5 positively loaded on PC2 and SE3 and SE4 negatively loaded on PC2. When examining weather parameters along with the wine data, some positive and negative associations can be observed (Figure 4C). In Year 1, both total and véraison to harvest GDD (GDD_T and GDD_V) were positively associated with green apple and thiol flavors, octanoic acid, and a*, while negatively associated with yellow color, VA, and phenylethyl alcohol.

In Year 2 (Figure 4B,D), 55.4% of the variance was captured in the first two dimensions. NW1 and NW3 were the only wines negatively loaded on PC1. NW1 was positively loaded on PC2 while NW3 was negatively loaded on PC2, along with NW2, SE2, and NC1. SE1 and SE3 were positively loaded on both dimensions. Contrary to Year 1, cumulative rainfall (Rainfall_T) and GDD for the whole growing season (GDD_T) and during fruit ripening (véraison to harvest; GDD_V) had varying associations with chemical data. Véraison to harvest GDD was positively associated with the volatile compounds phenylethyl alcohol and methionol, while negatively associated with various aromatic esters. Total GDD of the whole growing season was positively associated with 1-hexanol, isobutanol, and 3-hexen-1-ol, while it was negatively associated with malate and a number of esters, including isopentyl hexanoate, ethyl decanoate, and ethyl hexanoate. Rainfall from véraison to harvest (Rainfall_V) was positively associated with yellow color and phenylethyl 2-methylpropanoate. Total growing season rainfall was positively associated with various acetate and ethyl esters, while GDD_V was negatively associated with these compounds, as seen in their opposing locations on the plot (Figure 4D).

Regional trends were apparent and consistent from year to year when examining sensory data alone, but when combined with chemical and weather data, regional trends are not as well captured. In Year 1, both Rainfall_V and GDD_V were positively correlated to each other and appear to be independent from the total values of the respective parameter; however, in Year 2, GDD_V was negatively correlated with GDD_T, and the same correlation was seen for rainfall values. These trends were not surprising as there was not equal distribution of precipitation throughout the growing season among the regions (Table S1). For example, in Year 1, NW sites on average received 33% of total precipitation between véraison and harvest, while NE and SE sites received only 18% of total precipitation during this period. In Year 2, a similar pattern was observed, with approximately 26% of total precipitation being received from véraison to harvest at NW sites, while sites in the SE region received, on average, 8% of precipitation during this period. Distribution of GDD was more consistent across regions. In Year 1, 26–38% of total GDD was accumulated during the ripening period in all regions, while in Year 2, 24–31% of total GDD was accumulated during this period in all regions.

While there was variability in distribution of GDD and rainfall throughout the growing season, thiol aroma and flavor consistently associated with GDD_T and Rainfall_T in both years of study (Figure 4C,D). While volatile thiols have been studied extensively in other *vinifera* varieties, characterization of varietal thiols in Grüner V. has not been completed. With variation in thiol aroma and flavor in wines from different growing regions, along with its association with GDD and rainfall, there is potential that volatile thiols may contribute to characteristic Grüner V. aroma and flavor and can be further examined in future studies.

Heat accumulation (i.e., GDD) and rainfall are important weather factors that can influence final grape and wine quality, but these are only two parameters among a number of environmental and cultural factors that may be contributing to regional differences, of which were not fully captured in this study. Due to the large differences in weather patterns between regions in Pennsylvania and high variability in rainfall and GDD from season to season, it is difficult to determine the relationship between sensory and chemical parameters and environmental factors. A more robust examination of these factors in Pennsylvania’s wine regions in the future may provide insight into regional variation, similar to Harner et al. [52].

## 4. Conclusions

This study examined regional differences in Pennsylvania-grown Grüner V. wines across two vintages along with both sensory and instrumental analysis. This was the first study to examine regional differences in Pennsylvania using a controlled winemaking method, and also the first time Pennsylvania Grüner V. wines were examined by a trained sensory panel. Differences in orthonasal aroma, taste, mouthfeel, and in-mouth flavor were found between the wines when compared to each other as well as between wines from different growing regions. Sensory differences were also found for wines within the same growing region, specifically those located at the same commercial vineyard, suggesting mesoclimatic and soil factors might contribute to differences in wine sensory and chemical properties. While a number of aroma and flavor attributes were found to differ by region in Year 2, only yellow color was significantly different across regions in both years of study, with NW wines rated highest in yellow color in both years.

Regional sensory profiles were created for Grüner V. wines; however, regional differences were less clear when aspects were examined individually via instrumental methods. The presence or absence of precursor compounds is dependent on various conditions in the vineyard, of which were not examined fully in this study. While GDD and rainfall for the ripening period and entire growing season did show consistent associations with sensory and instrumental measures, namely in thiol aroma and thiol flavor, there are other important factors, both cultural and environmental, that may be contributing to regional differences that were not included in this study. Further work in examining Pennsylvania regionality with a more robust examination in cultural conditions may unveil additional explanations for these differences.

## Figures and Tables

**Table 1:** Reference standards used for the Descriptive Analysis (DA) sensory attributes for wines made in 2018 (Year 1) and 2019 (Year 2) All standards were created in Bota Box Pinot Grigio (Bota Box Vineyards, Manteca, CA) base wine unless otherwise noted.

Attribute	Reference Standard
Yellow Color	Rated on line scale with anchors “No Yellow” to “Very Yellow”
Haziness	Rated on line scale with anchors “No Haze” to “Very Hazy”
Green Apple	10.0 g fresh granny smith apple (Wegmans, State College, PA, USA) in 20 mL wine
Pear	25.0 g fresh Bartlett pear (Wegmans) in 25 mL wine
Other Fruit	10.0 g fresh nectarine + 10.0 g fresh peach (Wegmans) in 40 mL wine
	10 mL canned Fruit Cocktail juice (Wegmans) in 20 mL wine
Grape	3 halved fresh green grapes (Wegmans) in 20 mL wine
Citrus	3 × 2 cm fresh lemon peel (Wegmans) in 20 mL wine
	3 × 2 cm each fresh orange peel + grapefruit peel (Wegmans) in 40 mL wine
Floral	4 drops of Floral stock in 20 mL wine (Floral stock: 1 drop lavender essential oil (Aura Cacia, Norway, IA, USA) in 25 mL wine)
Earthy	1.0 g soil (Indoor Potting Mix, Miracle Gro Lawn Products Inc., Marysville, OH, USA) in 20 mL wine
Thiol	0.075 mL of 30 µM 4-methyl-4-mercaptopentan-2-one in 50 mL RO water
Canned Vegetable	2.5 mL canned pea juice + 2.5 mL canned green bean juice (Wegmans) in 20 mL wine
Rotten Egg	0.4 g hardboiled egg yolk in 20 mL wine
Sulfur	2 pinches potassium metabisulfite (Presque Isle Wine Cellars) in 25 mL RO water
Yeasty	2 pinches baker’s yeast (Fleischman’s ActiveDry Yeast, ACH Food Companies, Memphis, TN, USA) in 5 mL RO water
Oxidized	5 mL dry sherry (Taylor Wine Company, Canandaigua, NY, USA) in 20 mL wine
Chemical/Solvent	1 drop ethyl acetate (VWR International, Radnor, PA, USA) in 50 mL wine
Ethanol	10% (*v/v*) ethanol (Decon Labs, Inc., King of Prussia, PA, USA) solution
Sour	1.5 g/L tartaric acid (≥99.7%, Sigma-Aldrich, St. Louis, MO, USA) in RO water 2.0 g/L malic acid (≥99%, Sigma-Aldrich) in RO water
Sweet	20.0 g/L sucrose (Domino Foods, Inc., Yonkers, NY, USA) in RO water
Salty	2.0 g/L salt (Morton Salt, Chicago, IL, USA) in RO water
Bitter	0.8 g/L caffeine (Sigma-Aldrich) in RO water
Umami	5.0 g/L monosodium glutamate (B&G Foods, Inc., Parsippany-Troy Hills, NJ, USA) in RO water
Viscous/Thick	1.0 g/L carboxymethyl cellulose (Tic Gums, Belcamp, MD, USA) in RO water
Astringent	0.8 g/L alum (McCormick, Hunt Valley, MD, USA) in RO water
Warm/Hot	6% (*v/v*) ethanol (Decon Labs, Inc., King of Prussia, PA, USA) solution

## Data Availability

Available data is reported in the manuscript and supplementary materials.

## Supplementary Materials

The following are available online at https://www.mdpi.com/article/10.3390/foods10040825/s1. Table S1: Date of harvest, growing degree days (GDD), and rainfall quantified as véraison to harvest (_V) and total (_T) values for Year 1 and Year 2 vineyard sites, together with elevation and soil series details, Table S2: Significant differences between PA regions for Year 1 GV wines. Values that share the same letter within column are not significantly different according to Tukey’s post-hoc comparison (*p* < 0.05), Table S3: Juice chemistry and harvest data for Year 1 and Year 2 sites (TSS: total soluble solids; TA: titratable acidity; YAN: yeast assimilable nitrogen), Table S4: Significant differences between regions for Year 2 wines. Values that share the same letter within column are not significantly different according to Tukey’s post-hoc comparison (*p* < 0.05). Attributes that end in F indicate in-mouth flavor, A indicate aroma, T indicate taste, and MF indicate mouthfeel attributes, Table S5: Chemistry results for the wines (**a**)Year 1, (**b**) Year 2 (FR … fermentation replicate; RS … Residual sugar; TA … titratable acidity; LA … lactic acid; VA … volatile acidity), Table S6: Identified volatile compounds in wines in year 1, together with loadings for the first two dimensions of the PCA shown in Figure 4C, Table S7: Significant differences for volatile analysis between regions for Year 1. Internal standard equivalent values (μg/L) that share a letter in column are not significantly different according to Tukey’s post-hoc comparison (*p* < 0.05), Table S8: Identified volatile compounds in wines in year 2, together with loadings for the first two dimensions of the PCA shown in Figure 4D, Table S9: Significant differences for volatile analysis between regions for Year 2. Internal standard equivalent values (μg/L) that share a letter in column are not significantly different according to Tukey’s post-hoc comparison (*p* < 0.05), Table S10: ANOVA results with Region and Wine used as main factor. F-values in bold were significant (*p* < 0.05).
