# Supplementary material for: Influence of Region on Sensory and Chemical Profiles of Pennsylvania Grüner Veltliner Wines
Source: Foods. 2021 Apr 10;10(4):825. doi: 10.3390/foods10040825 (PMC8070092; doi:10.3390/foods10040825)
Supplement: Supplementary file 1 [file foods-10-00825-s001.pdf]

Table S1. Date of harvest, growing degree days (GDD), and rainfall quantified as véraison to harvest (_V) and total (_T) values for Year 1 and Year 2 vineyard sites, together with elevation and soil series details.

	NC1	NE1	NW1	NW3	NW2	SE1	SE2	SE3	SE4	SE5
Year 1										
Harvest Date	19-Sep	24-Sep	14-Sep	-	24-Sep	14-Sep	17-Sep	17-Sep	20-Sep	20-Sep
GDD_T	1680	1482	1595	-	1592	1668	1657	1611	1544	1733
GDD_V	653	437	378	-	481	409	426	417	424	480
Rainfall_T (mm)	784	782	452	-	379	626	768	696	895	553
Rainfall_V (mm)	212	139	168	-	107	76	194	100	210	76
Year 2										
Harvest Date	24-Sep	-	24-Sep	2-Oct	24-Sep	11-Sep	17-Sep	17-Sep	-	-
GDD_T	1645	-	1384	1459	1319	1612	1624	1556	-	-
GDD_V	439	-	413	488	404	346	446	378	-	-
Rainfall_T (mm)	468	-	499	509	427	414	679	655	-	-
Rainfall_V (mm)	77	-	123	132	115	24	63	67	-	-
Vineyard characteristics										
Elevation (m)	181	345	285		223	114	186	217	294	123
Soil series[a]	Elliber cherty silt loam	Oquaga channery silt loam	Venango silt loam		Harbor creek-Tyner complex	Conestoga silt loam	Berks-Weikert complex	Bedington-Berks complex	Berks channery silt loam	Manor loam

[a]Data sourced from the USDA National Resources Conservation Service (NRCS) Web Soil Survey, https://websoilsurvey.nrcs.usda.gov.

Table S2. Significant differences between PA regions for Year 1 GV wines. Values that share the same letter within column are not significantly different according to Tukey's post-hoc comparison ($p < 0.05$).

Region	Yellow Color	Haziness
NC	2.51a	2.73b
NE	4.20b	4.10b
NW	5.52c	1.29a
SE	2.87a	3.43b

Table S3. Juice chemistry and harvest data for Year 1 and Year 2 sites (TSS … total soluble solids; TA … titratable acidity; YAN … yeast assimilable nitrogen).

Site	Yield (gal)		TSS (°Brix)		pH		TA (g/L)		YAN (mg N/L)	
	Yr 1	Yr 2	Yr 1	Yr 2	Yr 1	Yr 2	Yr 1	Yr 2	Yr 1	Yr 2
NC1	13.5	12.25	14.2	20	3.22	3.29	7.409	6.731	248.2	139.3
NE1	13	-	16.4	-	3.25	-	7.708	-	221.2	-
NW1	16.2	18	18	14	3.06	3.04	7.331	9.514	57.6	105.5
NW2	13	15	19.6	19.2	3.41	3.11	5.683	8.545	128.2	113.5
NW3	-	8.25	-	16.6	-	3.07	-	8.143	-	107.6

SE1	0.75	19	14.2	18	3.56	3.69	6.446	5.362	98.9	201.0
SE2	11	11.25	16.4	19.6	3.36	3.51	6.152	6.809	177.3	137.5
SE3	13	13	15.6	19.6	3.52	3.75	6.428	7.118	241.1	157.8
SE4	9	-	16.4	-	3.28	-	6.256	-	216.0	-
SE5	3	-	16	-	3.48	-	5.542	-	261.2	-

Table S4. Significant differences between regions for Year 2 wines. Values that share the same letter within column are not significantly different according to Tukey's post-hoc comparison ($p < 0.05$). Attributes that end in F indicate in-mouth flavor, A indicate aroma, T indicate taste, and MF indicate mouthfeel attributes.

**Region**	**Yellow Color**	**Haziness**	**Green Apple_F**	**Citrus_F**	**Thiol_F**
NC	2.44a	2.90ab	3.39a	2.30a	1.15a
NW	4.28c	3.13b	4.01b	3.51b	1.30a
SE	3.50b	2.10a	3.73ab	2.86a	1.76b
**Region**	**Thiol_A**	**Sour_T**	**Sweet_T**	**Salty_T**	**Astringent_MF**
NC	1.36a	3.69a	3.67c	1.29a	3.83a
NW	1.73a	5.09c	2.36a	1.70b	4.76b
SE	2.37b	4.32b	2.98b	1.50ab	4.39ab

Table S5. Chemistry results for the wines **(a)** Year 1, **(b)** Year 2 (FR … fermentation replicate; RS … Residual sugar; TA … titratable acidity; LA … lactic acid; VA … volatile acidity).

	Wine	FR	% Alcohol	RS (g/L)	pH	TA (g/L)	Malate (g/L)	LA (g/L)	VA (g/L)	Free SO_2 (ppm)	Total SO^2 (ppm)
**(a)**	NC1	1	12.5	1.9	3.29	6.5	2.4	0	0.26	39	136
	NC1	2	12.4	2.3	3.27	6.4	2.4	0	0.27	45	141
	SE4	1	13.2	4.1	3.34	5.8	1.7	0	0.27	51	154
	SE4	2	13.2	4.5	3.31	5.9	1.7	0	0.3	42	144
	SE3	1	12.6	0.7	3.38	6.1	2.3	0	0.29	54	165
	SE3	2	12.7	0.8	3.39	6.1	2.3	0	0.3	49	163
	SE2	1	12.6	1	3.31	5.7	1.6	0	0.26	46	135
	SE2	2	12.6	0.8	3.29	5.7	1.4	0	0.29	42	132
	SE1	1	11.3	0.1	3.15	6.1	1.6	0	0.17	78	201
	SE1	2	11	0.1	3.18	6.0	1.6	0	0.24	87	196
	NW2	1	13.1	0.7	3.2	6.0	1	0.4	0.32	26	91
	NW2	2	13.1	0.7	3.2	6.0	1.1	0.4	0.31	48	126
	NE1	1	12.5	0.8	3.17	6.3	1.4	0.3	0.27	28	121
	NE1	2	11.9	0.9	3.17	6.6	1.3	0.8	0.27	35	124
	NW1	1	13.2	1	2.99	6.6	1.3	0.5	0.23	39	102
	NW1	2	13.2	1	3	6.6	1.2	0.5	0.25	42	106
	SE5	1	11.7	0.5	3.26	6.0	1.6	0	0.33	48	138
	SE5	2	11.6	0.5	3.21	6.4	1.6	0.4	0.3	49	148
**(b)**	NC1	1	12.6	9.6	3.21	6.9	2.1	0.2	0.24	15	92
	NC1	2	12.8	8.3	3.24	6.4	1.9	0.0	0.29	16	92
	SE3	1	12.9	2.3	3.12	7.8	2	0.3	0.3	11	103
	SE3	2	13.2	2.3	3.17	7.7	2.6	0.2	0.27	13	107
	SE2	1	12.5	4.2	3.10	7.0	2	0.2	0.18	13	104
	SE2	2	13.0	3.2	3.29	7.2	2.2	0.0	0.09	10	101
	SE1	1	12.4	1.0	3.18	7.2	1.9	0.4	0.26	10	70
	SE1	2	12.6	0.9	3.20	7.2	2	0.3	0.31	9	71
	SE1	3	12.6	0.8	3.16	7.3	1.9	0.5	0.28	7	68
	NW2	2	12.3	3.4	3.01	7.6	2.5	0.0	0.31	11	79
	NW2	1	12.4	2.6	3.05	8.2	2.6	0.3	0.27	10	80
	NW1	1	11.8	0.8	2.81	9.2	2.6	0.5	0.23	7	40
	NW1	2	11.9	0.6	2.89	9.2	2.8	0.4	0.11	4	36
	NW3	1	12.8	3.1	3.05	7.8	2.1	0.0	0.1	11	56
	NW3	2	11.7	2.3	3.05	8.0	2.1	0.1	0.15	11	54

Table S6. Identified volatile compounds in wines in year 1, together with loadings for the first two dimensions of the PCA shown in **Figure 4C**.

Volatile Compound	Dim. 1	Dim. 2	Volatile Compound	Dim. 1	Dim. 2
Sulfur Dioxide	-0.19	-0.01	1-Propanol-3-Ethoxy	0.97	0.02
Ethyl Acetate	0.28	0.36	Methyl Octanoate	0.85	0.24
Ethanol	-0.62	0.23	Ethyl Octanoate	-0.82	-0.09
Decane	-0.75	0.33	2,3-Butanediol	0.93	-0.01
1-Propanol	0.93	0.08	Methyl Decanoate	0.94	0.14
1-Butanol-2-Methyl-Acetate	0.34	-0.04	Ethyl Decanoate	-0.66	-0.39
Ethyl 2-Butenoate	-0.03	-0.16	2-Phenyl Acetate	-0.01	-0.85
Pentyl Acetate	0.91	0.11	Hexanoic Acid	0.94	-0.02
Methyl Hexanoate	0.92	0.11	Phenylethyl Alcohol	-0.55	0.71
1-Butanol-3-Methyl	0.04	0.61	1-Dodecanol	0.92	0.18
Ethyl Hexanoate	-0.85	-0.14	Octanoic Acid	0.83	-0.42
Hexyl Acetate	-0.65	-0.66	Decanoic Acid	0.92	0.04
4-Hexen-1-ol Acetate	-0.52	-0.69	9-Decenoic Acid	0.92	0.18
2-Hexen-1-ol Acetate	-0.36	-0.70	Ethyl Hydrogen Succinate	0.91	0.18
1-Pentanol-3-Methyl	0.92	0.26	Dodecanoic Acid	0.93	0.19
Ethyl lactate	0.95	0.21	Ethyl-9-Decenoate	0.95	-0.04
1-Hexanol	0.01	0.63			

Table S7. Significant differences for volatile analysis between regions for Year 1. Internal standard equivalent values (μg/L) that share a letter in column are not significantly different according to Tukey's post-hoc comparison ($p < 0.05$).

Region	Ethyl hexanoate	Hexyl acetate	2-Hexen-1-ol acetate	1-Hexanol	Ethyl octanoate	Phenylethyl alcohol
NC	3.02b	1.323b	0.0205b	0.442a	13.2ab	10.1ab
NE	2.18ab	0.896a	0.0120ab	0.606b	10.0ab	10.7ab
NW	2.80b	0.895a	0.0114a	0.535ab	13.1b	13.5b
SE	2.15a	1.033a	0.0149ab	0.483ab	9.4a	10.6a

Table S8. Identified volatile compounds in wines in year 2, together with loadings for the first two dimensions of the PCA shown in **Figure 4D**.

Volatile Compound	Dim. 1	Dim. 2	Volatile Compound	Dim. 1	Dim. 2
Carbon dioxide	0.31	-0.76	Methyl Octanoate	-0.78	0.21
Sulfur dioxide	0.68	-0.49	Acetic Acid	0.78	0.11
Ethyl Acetate	0.55	0.78	Isopentyl Hexanoate	-0.44	-0.1
Isobutyl Acetate	0.26	0.69	Octyl Acetate	0.16	-0.05
Ethyl Butanoate	-0.60	0.13	Methyl Decanoate	-0.5	0.33
Propanol	0.52	0.61	Butanoic Acid	-0.94	0.13
Butyl Acetate	0.40	0.86	Ethyl Decanoate	-0.4	-0.01
2-Methyl Propanol	0.65	0.38	Ethyl 3-Methylbutyl Octanoate	-0.65	0.03
Methyl 3-Butanoate	0.19	0.73	Ethyl-9-decenoate	0.15	-0.6

Ethyl-2-Butenoate	0.14	0.73	Methionol	0.18	-0.8
Pentyl Acetate	0.42	0.82	2-Phenylethyl Acetate	0.48	0.14
Methyl hexanoate	-0.8	0.16	Hexanoic Acid	-0.86	0.06
D-Limonene	0.84	-0.33	Ethyl Dodecanoate	-0.46	0.09
3-Methyl-Butanol	0.58	-0.11	3-Methylbutyl pentadecanoate	-0.39	0.07
Ethyl Hexanoate	-0.64	-0.04	Phenylethyl Alcohol	-0.12	-0.9
Hexyl Acetate	0.63	0.71	1-Dodecanol	0.34	-0.39
Ethyl-4-hexenoate	0.46	0.54	Octanoic Acid	-0.93	-0.07
4-Hexen-1-ol Acetate	0.67	0.57	Ethyl 3-hydroxytridecanoate	-0.57	0.56
4-Methyl-1-Pentanol	0.27	-0.74	2-Methoxy-4-vinylphenol	0.41	0.01
2-Hexen-1-ol Acetate	0.52	0.63	Decanoic Acid	-0.48	0.31
3-Methyl-1-Pentaol	0.34	-0.39	β-damascenone	-0.64	0.22
Ethyl 2-Hexenoate	0.71	0.64	3-Ethyl-4-methylpentanol	0.44	0.62
Ethyl Lactate	-0.44	0.53	2-Hexen-1-ol Acetate	0.15	0.81
1-Hexanol	0.71	0.41	2-Hexenoic Acid	0.52	0.75
3-Hexen-1-ol (Z)	0.59	0.31	Phenethyl 2-methylpropanoate	-0.41	-0.41
3-Ethoxy propanol	0.14	0.85			

Table S9. Significant differences for volatile analysis between regions for Year 2. Internal standard equivalent values (μg/L) that share a letter in column are not significantly different according to Tukey's post-hoc comparison ($p < 0.05$).

**Region**	**Ethyl acetate**	**Isobutyl acetate**	**Ethyl butyrate**	**1-Propanol**	**Butyl acetate**
NC	2.03a	0.1004ab	0.476ab	0.0816a	0.01116a
NW	2.02a	0.0846a	0.518b	0.0853a	0.00989a
SE	2.37b	0.1301b	0.469a	0.1304b	0.01986b
**Region**	**Isobutanol**	**Isoamyl acetate**	**Amyl acetate**	**Methyl hexanoate**	**1-Hexanol**
NC	0.247a	10.08ab	0.0234a	0.0219a	0.537a
NW	0.248a	9.24a	0.0225a	0.0456c	0.486a
SE	0.276b	12.06b	0.0280b	0.0335b	0.644b
**Region**	**3-Hexen-1-ol**	**Methyl octanoate**	**Acetic acid**	**Octyl acetate**	**2-Hexen-1-ol**
NC	0.0241b	0.164a	0.253a	0.185b	0.00703a
NW	0.0117a	0.301c	0.234a	0.150a	0.00682a
SE	0.0286b	0.239b	0.314b	0.166ab	0.00930b

Table S10. ANOVA results with Region and Wine used as main factor. F-values in bold were significant ($p < 0.05$).

	**Gallic Acid Yr 1**	**Catechin Yr 1**	**Epicatechin Yr 1**	**Gallic Acid Yr 2**	**Catechin Yr 2**	**Epicatechin Yr 2**
DF - Region	3	3	3	2	2	2
F-Value - Region	0.3473	**5.9789**	**14.506**	0.9079	0.2686	0.5088
DF - Wine	8	8	8	6	6	6
F-Value - Wine	**19.591**	**105.84**	**80.577**	**129.27**	**127.56**	**106.7**
